# Editorial: Microorganisms and their derivatives for cancer therapy

**DOI:** 10.3389/fbioe.2023.1137341

**Published:** 2023-01-13

**Authors:** Yunlei Zhang, Lígia R. Rodrigues, Zhenping Cao, Juanjuan Li

**Affiliations:** ^1^ Department of Respiratory and Critical Care Medicine, The Affiliated Jiangning Hospital of Nanjing Medical University, Nanjing, China; ^2^ Department of Biomedical Engineering, School of Biomedical Engineering and Informatics, Nanjing Medical University, Nanjing, China; ^3^ CEB—Centre of Biological Engineering, University of Minho, Braga, Portugal; ^4^ Shanghai Key Laboratory for Nucleic Acid Chemistry and Nanomedicine, Institute of Molecular Medicine, State Key Laboratory of Oncogenes and Related Genes, Shanghai Cancer Institute, Renji Hospital, School of Medicine, Shanghai Jiao Tong University, Shanghai, China; ^5^ School of Life Sciences, Hainan University, Haikou, China

**Keywords:** microorganism, bacteriotherapy, bacteriolytic therapy, drug delivery, bacterial products, cancer therapy

Cancer remains an unsolved and challenging problem. In 1890, Dr. William Bradley Coley attempted to use a mixture of dead microbes to treat cancers (Dobosz and Dzieciatkowski, 2019; Liu et al., 2022), establishing the foundation of bacteria-mediated cancer therapy. Given the recent advances in the study of the human microbiome that revealed its crucial role in tumorigenesis, development, therapy, and prognostic evaluation, additional research efforts on cancer microbial therapies have been conducted (Kurtz et al., 2019; Feng et al., 2022), with new findings supporting the potential role of bacteriolytic therapy in cancer. Our Special Research Topic aimed at exploring the trends and recent advances on the use of microorganisms and their derivatives for cancer therapy, on new anticancer agents, new genetic engineering techniques, and synthetic or new identified bacteria, which could be used for cancer monotherapy or adjuvant therapy, as well as understanding the mechanisms underlying their anticancer effects.

Overall, a total of five manuscripts comprising three original research articles and two review papers were published within this Special Research Topic in 11 months. The research papers addressed new anticancer agents (new anticancer cytotoxic compounds from a probiotic microorganism; new identified tumor-targeting bacterium, and a genetically engineered immunotherapeutic bacterium), and the reviews were focused on bacteriolytic therapy. Previously, bacterial derivatives, such as CpG DNA (M. Liu et al., 2019) and flagellin B (Zheng et al., 2017), have been reported to trigger strongly non-specific immunotherapy responses in cancer. Encouragingly, dozens of clinical trials using CpG DNA in various cancers are being conducted (Ma et al., 2018). Recently, given the advances on DNA sequencing, genetic operation and microorganism culture techniques, especially the heterogeneous expression of large gene clusters from non-culturable microbes, several functional substances that greatly contribute to drug libraries of anticancer agents and anti-bacteria have been successfully produced (Liu et al., 2022). In Zhang’s work, the cell-free culture supernatant of *Lactiplantibacillus plantarum* YT013 isolated from serofluid dish showed remarkable inhibition of different tumors, suggesting *Lactiplantibacillus plantarum* YT013 as a good candidate for anticancer therapies. Extracts from *Lactipantibacillus plantarum* YT013 further enriched the anticancer drug library, given that the effective compounds can be identified and analyzed clearly. Several well-known bacteria, including *Salmonella typhimurium*, *Escherichia coli* Nissle 1917, *Clostridium difficile*, *Listeria monocytogenes*, and *Bifidobacterium* have been widely used as tumor-targeting bacteria (Liu et al., 2022). However, additional new tumor-targeting bacterium could not be easily found from millions of published papers. Zhang et al. identified a new tumor-targeting bacterium, *Xenorhabdus stockiae*, isolated from a soil sample *via* an entrapment method using *Galleria melonella* nematodes. Notably, the newly identified bacterium reported in this Special Research Topic not only was found to colonize solid tumors but also to produce cytotoxic compounds and significantly inhibit the growth of B16 melanoma, thus exhibiting excellent characteristics of a tumor-targeting bacterium. It is expected that the authors further develop their research using this bacterium or provide it to other researchers towards a deeper exploitation of its potential and eventually bringing it into clinical trials. Besides the use of newly identified anticancer bacterium, the genetic modification of the currently existing tumor-targeting bacteria to express cytotoxic drugs or some key factors is also an effective strategy to regulate tumor growth, migration, drug resistance, and drug tumor penetration to restrain or prevent tumor growth. For example, Xu et al. genetically modified the widely used *Salmonella* to express IFN-γ to activate tumor-infiltration of M1-like macrophage and CD^4+^ and CD^8+^ T cells that further eradicate tumors.

Additionally, two papers summarized and discussed the recent studies on bacteriolytic therapy. Geng et al. mainly focused on the description of bacteria-mediated cancer therapy, ranging from the mechanisms underlying why bacteria target and colonize solid tumors to the applications of the bacteria for cancer therapy with or without genetic modification. Wang et al. focused on surface modifications on tumor-targeting bacteria using various materials, thus contributing to their targeting abilities, photothermal effect, immunotherapy, among other activities and potentialities. The authors also put forward some new concepts related to the development of bacteriolytic therapy based on the tumor specific microenvironment, new genetic modification techniques, nanomaterials, sources of non-culturable microbes and their derivatives.

Despite these advancements, the use of microorganisms and their derivatives to treat cancers is still in the infancy stage and hindered by potential pathogenicity. Of note, most studies mainly focus on the use of microbes or compounds to directly kill cancer cells. However, with the advent of the immunotherapy era, we should envision that using single or multiple strains to adjuvant chemotherapy, immunotherapy, radiation therapy or other curative strategies through regulating intestinal flora, altering immunosuppressive microenvironment, secreting useful substances could cross local laws and regulations, which allow clinical trials to use the wild bacteria for disease treatment. Furthermore, using derivatives of these microorganisms for cancer non-specific immunotherapy will make an important contribution to cancer treatment that current drugs are not being able to address.
